# Drug Reaction With Eosinophilia and Systemic Symptoms (DRESS) Syndrome Caused by Apalutamide: A Case Presentation

**DOI:** 10.7759/cureus.41687

**Published:** 2023-07-11

**Authors:** Gabriella Martin, Evan Lambert, Gordon K Wang

**Affiliations:** 1 Medical School, Nova Southeastern University Dr. Kiran C. Patel College of Osteopathic Medicine, Fort Lauderdale, USA; 2 Family Medicine, Nova Southeastern University Dr. Kiran C. Patel College of Osteopathic Medicine, Fort Lauderdale, USA

**Keywords:** apalutamide, drug reaction, eosinophilia, rash, prostate cancer, side effects, medication, dress

## Abstract

Drug reaction with eosinophilia and systemic symptoms (DRESS) is a severe drug reaction that is triggered several weeks after the start of a new medication. This syndrome presents with a variety of clinical symptoms, specifically a manifestation involving a fever followed by a severe rash. A variety of medications are known to trigger DRESS, with the most common being anticonvulsants and allopurinol. Here, we discuss the case of a medication, apalutamide, that caused DRESS in our patient. Early recognition and abrupt discontinuation of the medication is required for the management of this syndrome and to minimize morbidity and mortality.

## Introduction

Drug reaction with eosinophilia and systemic symptoms (DRESS) is a multi-organ systemic drug reaction that has the potential to be fatal. DRESS is more common in females, with symptoms occurring two to six weeks after medication use and can last up to months after medication discontinuation [1-4]. The classic presentation of patients with DRESS syndrome is fever, rash, lymphadenopathy, hematological abnormalities, and involvement of at least one organ system [5]. The rash is often polymorphic with a variety of skin manifestations, including maculopapular exanthema, lichenoid, exfoliative, urticarial, purpuric, eczematous, and pustular lesions [6]. The rash is accompanied by dermal edema and inflammation. In addition, there is a lack of blistering, which differentiates it from Steven-Johnson syndrome/toxic epidermal necrolysis (TEN) [7].

In DRESS patients, aberrant T helper type 2 (Th2) cells are recruited to the cell. Interleukin (IL)-5 is produced by the Th2 cells that cause the activation and migration of eosinophils to the peripheral blood and tissues [8]. Th2-lymphocytes and cluster of differentiation 8+ (CD8+) cells cause a type IVb hypersensitivity reaction to the skin and internal organs [9]. The most common agents that cause DRESS syndrome are the anticonvulsant drugs and allopurinol. There are also several reports of DRESS being caused by antimicrobial agents, such as vancomycin, sulfonamides, beta-lactams, and minocycline [10]. However, other medications have the potential to cause this severe rash accompanied with systemic symptoms. Apalutamide is an androgen receptor inhibitor that is used for the treatment of prostate cancer to prevent metastasis. Adverse effects of a skin rash was reported in 23.8% of patients treated with apalutamide [11]. Understanding how to clinically diagnose a patient with DRESS syndrome is important in management and treatment. In this case presentation, we present the case of a 74-year-old male who was found to be taking apalutamide for his recent diagnosis of prostate cancer. He developed DRESS and his management is discussed below.

## Case presentation

A 74-year-old male patient presented to the clinic with a pruritic, erythematous maculopapular rash on his hands, arms, and shoulders, bilaterally. The patient explained that the rash began on his right hand four days prior. The rash had progressed upward toward his neck. Upon questioning, the patient denied having tried any new soaps or detergents. In addition, the patient had not been in the sun recently. However, the patient was started on a new medication in January.

The patient was diagnosed with prostate cancer in January of 2023 and was started on apalutamide. The patient also has a past medical history of hypertension, diagnosed in 2022. In addition to apalutamide, the patient was taking Advil 200 mg orally as needed for pain, amlodipine 5 mg orally once a day, lisinopril 10 mg orally once a day, vitamin B12 1000 mcg orally once every other day, and vitamin D3 50 mcg orally once a day. The patient was advised to discontinue apalutamide immediately until the rash is gone. He was also advised to follow up with oncology regarding his treatment plan for his prostate cancer. He was prescribed a tapering dose of prednisone 10 mg orally: four times a day for three days, then three times a day for three days, then twice a day for three days, and then daily for three days. 

**Figure 1:**
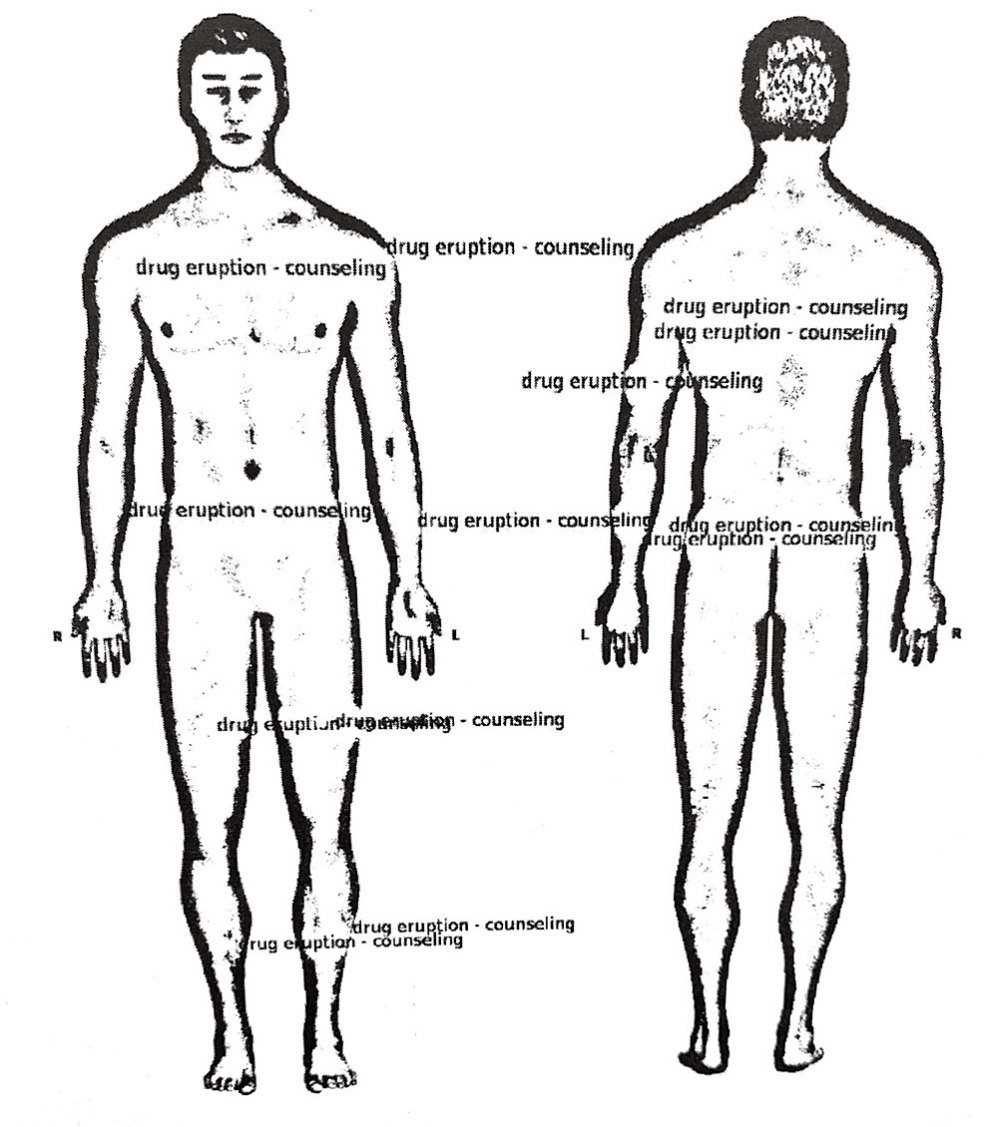
Rash distribution Maculopapular rash present on the patient's hands, arms, shoulders, trunk, and legs bilaterally. Figure received from South Florida Skin Center.

**Figure 2:**
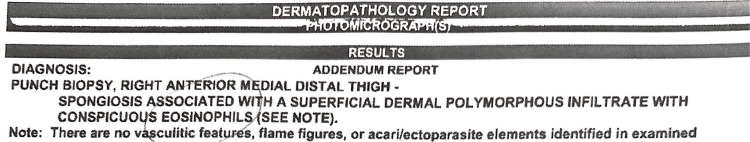
Punch biopsy results

The patient presented back to the clinic two weeks later with a diffuse erythematous maculopapular rash extending to his face, trunk, and legs, bilaterally (Figure 1). The patient started having fever, chills, myalgias, swelling of the arms and legs, and enlarged cervical lymph nodes. In addition, the rash began to desquamate. Labs were drawn and revealed a white blood count (WBC) of 16.1 thousand/uL (normal range of 3.8-10.8 thousand/uL), absolute eosinophils of 6360 cells/uL (normal range of 15-500 cells/uL), and alkaline phosphatase of 47 U/L (normal range of 35-144 U/L). The punch biopsy results came back and showed spongiosis with superficial dermal polymorphous infiltrate with eosinophils (Figure 2). At this visit, the patient was prescribed famotidine 20 mg orally twice a day, levocetirizine 5 mg orally twice a day, and prednisone 10 mg orally daily.

Two days later, the patient presented to the clinic for follow-up. The rash was still present on the entire body. The skin was scaly and peeling with some subcutaneous edema present. The patient had chills but was afebrile. At this point, the patient’s prednisone was increased to 20 mg orally three times a day. He continued taking famotidine 20 mg orally twice a day and levocetirizine 5 mg orally twice a day. 

At the end of March, the patient was seen in follow-up for his rash. He no longer had any swelling, chills, or fever. He only had mild itching, and the rash had subsided except for some peeling. The skin was also less inflamed. At this time, the patient was advised to decrease the prednisone dose gradually. He also discontinued famotidine and levocetirizine at this time.

## Discussion

DRESS is a rare drug reaction that can occur after exposure to certain medications. Apalutamide, an androgen receptor inhibitor used for the treatment of prostate cancer and prevention of metastasis, has been reported to cause skin rash, but few cases have been reported of the development of DRESS syndrome due to apalutamide [11]. Our patient’s use of apalutamide coincides with his onset of DRESS, as his symptoms began approximately four to six weeks after starting the medication. Our case contributes further evidence for clinicians to be aware of adverse effects when prescribing apalutamide. 

DRESS presentation can vary by individual as they can present with urticaria-like plaques, vesicles, pustules, fever, edema, lymphadenopathy, leukocytosis, eosinophilia, hepatitis, nephritis, pancreatitis, and myocarditis. The rash commonly involves over 50% of the body surface and with severe edema [5]. The Registry of Severe Cutaneous Adverse Reactions (RegiSCAR) scoring system, developed to diagnose cutaneous adverse reactions, can be used to accurately define the diagnostic criteria for DRESS (Table 1) [1,12].

**Table 1:** The Registry of Severe Cutaneous Adverse Reactions (RegiSCAR) scoring system to diagnose cutaneous adverse reactions Final scoring: <2: no case; 2-3: possible case; 4-5: probable case; >5: definite case ANA: antinuclear antibody; HAV/HBV/HCV: hepatitis A virus/hepatitis B virus/hepatitis C virus

Symptoms	No	Yes	Unknown
Fever (>38.5^o^C)	-1	0	--1
Enlarged lymph nodes	0	1	0
Eosinophilia, cells/uL			
700-1499 or 10%-19.9%	0	1	0
≥1500 or ≥20%	0	2	0
Skin rash (% body surface area)			
Extent: >50%	0	1	0
Skin rash suggesting DRESS	-1	1	0
Biopsy suggesting DRESS	-1	0	0
Organ involvement			
One	0	1	0
Two or more	0	2	0
Resolution ≥15 days	-1	0	-1
Exclusion of ≥three other potential causes (ANA, blood culture, HAV/HBV/HCV, Chlamydia/mycoplasma	th0	1	0

In this case, the patient had a final score of 5 in the RegiSCAR scoring system due to enlarged lymph nodes (one point), eosinophilia ≥1500 (two points), and skin rash suggesting DRESS with extent over 50% of the body surface area (two points) for a total of five points. 

Treatment of DRESS involves first discontinuing the causative drug, and it has been suggested that earlier withdrawal can lead to better prognosis [13]. After discontinuation, patients can be treated with supportive care if symptoms are mild, however, severe symptoms can be treated with a high dose corticosteroid as was done with our patient. There is also evidence that cyclosporine can be an effective treatment for DRESS and may perform equally as well as steroids [14].

## Conclusions

DRESS is an uncommon reaction that can be difficult to diagnose due to the time associated between symptoms appearing and the start of the offending agent. Patients who present with DRESS after starting apalutamide should be monitored closely for end-organ damage and have treatment initiated quickly to reduce morbidity and mortality. This case highlights the importance of recognizing clinical findings of DRESS and discontinuing the medication immediately to reduce life-threatening consequences.
